# A Rare Recurrence of Guillain-Barré Syndrome in a 72-Year-Old Female Presenting With Nausea and Vomiting Only

**DOI:** 10.7759/cureus.85283

**Published:** 2025-06-03

**Authors:** Omar Demachkie, Andres Saucedo, Everardo Tafolla, Sivarama K Kotikalapudi

**Affiliations:** 1 College of Osteopathic Medicine, William Carey University College of Osteopathic Medicine, Hattiesburg, USA; 2 Orthopedic Surgery, William Carey University College of Osteopathic Medicine, Hattiesburg, USA; 3 Neurology, William Carey University College of Osteopathic Medicine, Hattiesburg, USA; 4 Internal Medicine, Merit Health Wesley Hospital, Hattiesburg, USA

**Keywords:** autonomic variant guillain-barré syndrome (a-gbs), covid-19, guillain-barré syndrome (gbs), intractable nausea and vomiting, recurrent gbs

## Abstract

Guillain-Barré syndrome (GBS) is a neurological disorder that typically presents with ascending paralysis. However, atypical presentations can complicate the diagnostic process. We describe the case of a 72-year-old female with a prior history of GBS who presented with intractable nausea and vomiting without classic motor symptoms. This case highlights the diagnostic challenges of recognizing atypical GBS recurrence, particularly in elderly patients, and shows the importance of considering non-motor symptoms in patients with a previous history of GBS.

## Introduction

Guillain-Barré syndrome (GBS) is an immune-mediated disorder affecting the peripheral nervous system, leading to acute polyradiculoneuropathy. Most cases follow infections, commonly *Campylobacter jejuni*, cytomegalovirus (CMV), Epstein-Barr virus (EBV), *Mycoplasma pneumoniae*, and influenza virus [1]. Although GBS is traditionally considered a monophasic illness, recurrence occurs in approximately 1-3% of cases, typically emerging months to years after the initial episode [2].

Autonomic dysfunction is a common feature, occurring in up to 41.53% of cases [3]. Recent studies have shown that vagus nerve enlargement can occur in patients with GBS, particularly among those exhibiting autonomic dysregulation [4]. Vagal involvement may impair parasympathetic control of gastrointestinal (GI) motility, contributing to symptoms such as gastroparesis, nausea, and vomiting, especially in cases where routine GI workup is unremarkable.

We present the case of an elderly patient with a history of GBS who developed recurrent symptoms manifesting exclusively as intractable nausea and vomiting, without new-onset weakness. This presentation occurred two years after the initial GBS diagnosis. The case highlights the importance of recognizing non-motor manifestations of GBS recurrence, particularly autonomic symptoms, which can mimic primary GI pathology and delay appropriate diagnosis and treatment.

## Case presentation

A 72-year-old female with a history of GBS (2023), hypothyroidism, hypertension, hyperlipidemia, gastroesophageal reflux disease (GERD), hiatal hernia, and major depressive disorder presented to the emergency department (ED) with intractable nausea and vomiting for three days, along with an inability to tolerate oral intake. She reported up to eight episodes of vomiting in a single day. She denied fever, abdominal pain, diarrhea, or hematemesis. Physical examination was unremarkable, including a normal GI exam - the abdomen was soft, non-tender, non-distended, with normal bowel sounds and no organomegaly. However, dry skin was noted on examination, suggestive of dehydration.

Of note, the patient was recently discharged after an admission for similar symptoms four days prior. During that hospitalization, her symptoms had temporarily improved with diazepam and steroids. Extensive GI workup, including upper and lower endoscopy and abdominal imaging, was unremarkable, and her gastroenterologist ruled out a primary GI pathology.

**Figure 1:**
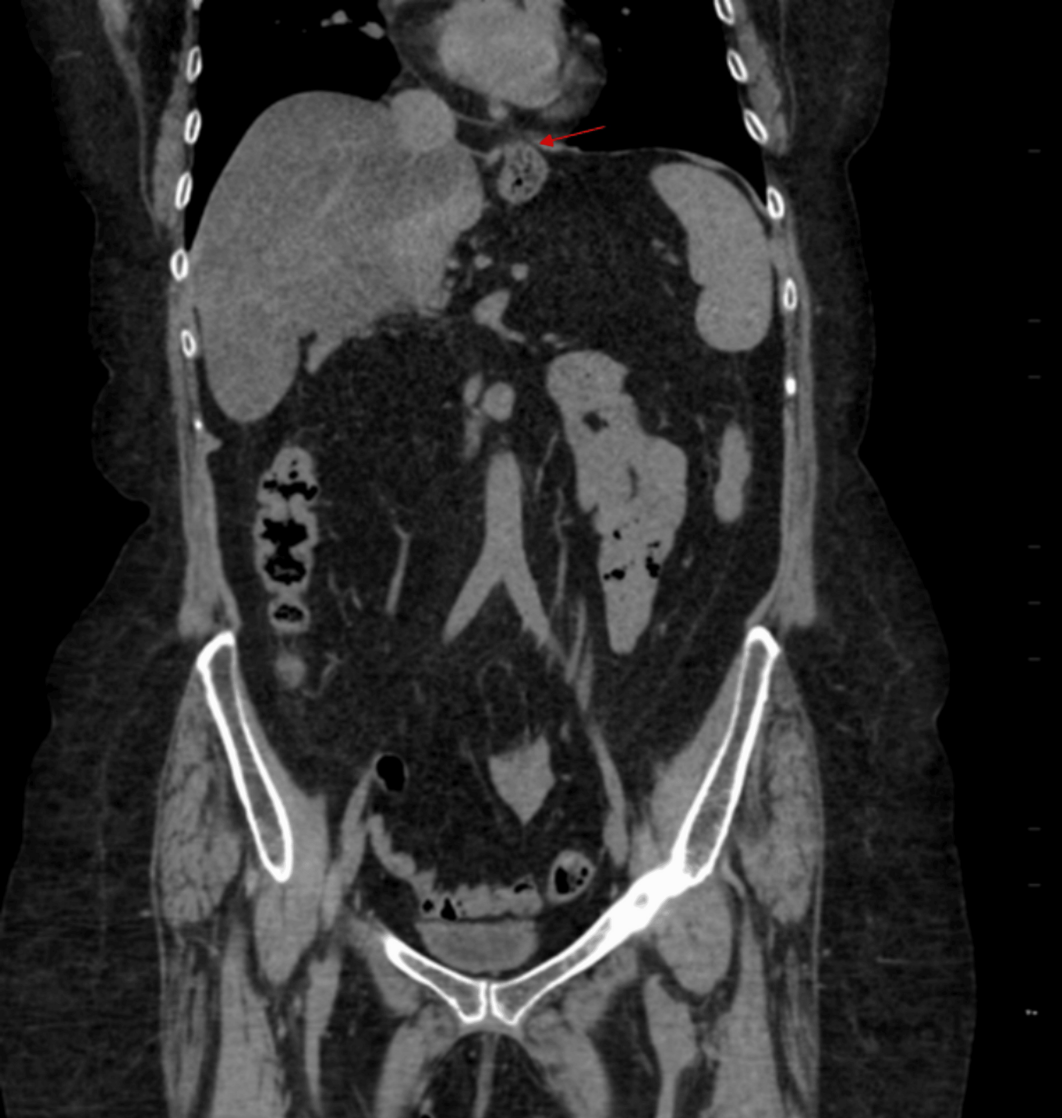
CT abdomen Moderate-sized hiatal hernia (indicated by the red arrow). No acute bowel obstruction. No pneumoperitoneum. No CT evidence of acute colonic diverticulitis. CT: computed tomography

**Figure 2:**
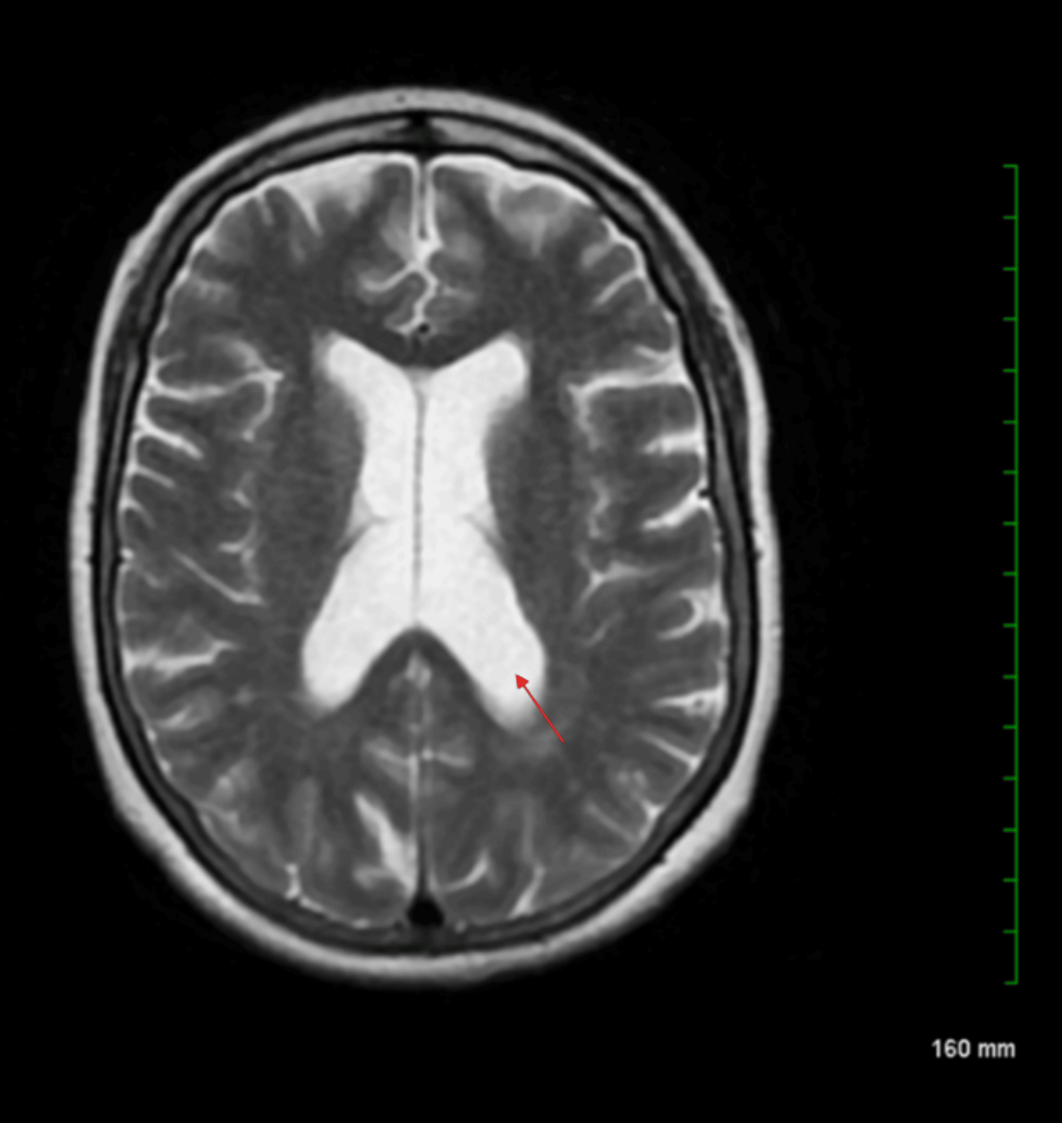
MRI brain without contrast No acute intracranial abnormality. Mild central cortical atrophy with associated ventricular enlargement (indicated by the red arrow). Trace scattered white matter disease noted bilaterally. MRI: magnetic resonance imaging

The patient was found to have hypokalemia (potassium (K^+^) 2.4 mmol/L), which was repleted with intravenous (IV) potassium chloride. Her complete metabolic panel (CMP) showed no evidence of renal or hepatic dysfunction. Lipase was 22 U/L, lowering suspicion for acute pancreatitis. The complete blood count (CBC) demonstrated a normal white blood cell (WBC) count with no signs of infection and stable hemoglobin and hematocrit, ruling out anemia (Table 1). Computed tomography (CT) of the abdomen/pelvis revealed fatty liver infiltration, fatty atrophy of the pancreas, and a hiatal hernia, with no acute pathology (Figure 1). Magnetic resonance imaging (MRI) of the spine lumbar without contrast showed multilevel central canal stenosis without significant nerve root impingement. MRI of the brain without contrast showed no acute intracranial abnormality, while mild central cortical atrophy with associated ventricular enlargement and trace scattered white matter disease were noted bilaterally (Figure 2).

**Table 1:** General chemistry and thyroid function panel results on day of admission WBC: white blood cell; ALT: alanine aminotransferase; AST: aspartate aminotransferase; TSH: thyroid-stimulating hormone

Labs	Results	Reference Range
WBC count	5.60	4.0 - 11.0 x 10^9^/L
Platelet count	171	130 - 300 x 10^3^/µL
TSH	1.665	0.4 - 4.0 mIU/L
Sodium	141	135 - 145 mmol/L
Potassium	2.4	3.5 - 5.0 mmol/L
Chloride	100	98 - 106 mmol/L
Carbon dioxide	32	22 - 29 mmol/L
Blood urea nitrogen	6	7 - 20 mg/dL
Creatinine	0.57	0.6 - 1.3 mg/dL
Anion gap	9.0	8 - 16 mmol/L
Alkaline phosphatase	81	44 - 147 IU/L
AST	28	10 - 40 IU/L
ALT	7	7 - 56 IU/L
Bilirubin total	0.90	0.1 - 1.2 mg/dL
Albumin	3.3	3.4 - 5.4 g/dL
Protein total	5.3	6.0 - 8.3 g/dL
Lipase	22	10 - 140 U/L

Given the recurrence of these symptoms without a clear GI, metabolic, cardiac, or infectious cause, she was admitted for further evaluation.

Despite temporary improvement with diazepam (Valium) during her first hospitalization, the patient reported that, during this admission, diazepam not only failed to relieve her nausea but actually worsened it. On day 2 of admission, her daughter mentioned finding a case study online describing GBS presenting solely with nausea and vomiting. The case involved a 40-year-old female and noted that a rare variant of GBS can affect primarily the autonomic nervous system, with minimal or no somatic motor involvement [5].

Her initial diagnosis of GBS was made based on clinical suspicion following ascending paralysis after a viral illness (COVID-19 infection), and was subsequently confirmed by nerve conduction studies and electromyography. The patient was treated with intravenous immunoglobulin (IVIG) for GBS.

Given the patient’s persistent symptoms, lack of improvement, and history of GBS, there was growing concern for a recurrent autonomic-dominant form of GBS. Therefore, the decision was made to initiate IVIG therapy on day 2. Following the initiation of IVIG, the patient experienced marked improvement, with resolution of her nausea and vomiting. She was able to tolerate oral intake and her medications without difficulty, supporting a positive response to IVIG treatment. By day 4 of IVIG therapy, the patient continued to show significant improvement, requiring only occasional doses of promethazine for residual nausea control. By day 5, she was able to tolerate a regular diet without difficulty and was discharged from the hospital in a medically stable condition.

## Discussion

While GBS typically presents with generalized motor weakness and frequent autonomic involvement, there is a rare variant that primarily affects the autonomic nervous system with minimal somatic motor symptoms. Due to the rarity of such cases, there is limited literature on recurrent GBS presenting solely with atypical symptoms. This variant often has a rapid onset, particularly in younger individuals, and generally carries a favorable prognosis over two to three years. However, recognition of this form is often difficult and delayed, leading to missed opportunities for timely treatment. The clinical presentation - commonly involving cardiac and GI symptoms - should prompt clinicians to consider this diagnosis in patients with otherwise unexplained autonomic dysfunction [5].

A study of 234 recurrent GBS cases found that 11 patients (representing 4.7%) experienced recurrences with atypical symptoms, often preceded by respiratory or GI illnesses [6]. As mentioned in the introduction, the proposed mechanism behind nausea and vomiting in recurrent GBS involves autonomic dysfunction with vagus nerve involvement, which may impair parasympathetic control of GI motility [4]. 

Diazepam (Valium) has been studied for its potential to alleviate GI spasms, particularly in patients with functional GI disorders where anxiety may be a contributing factor. In a double-blind, crossover clinical trial involving 28 patients with functional GI symptoms, diazepam (5 mg twice daily) and lorazepam (1 mg twice daily) were compared to placebo. Both benzodiazepines significantly outperformed the placebo in reducing GI symptoms, supporting their effectiveness in managing anxiety-related GI spasms [7].

Diazepam’s ability to enhance gamma-aminobutyric acid (GABA) activity may explain the patient’s initial symptom relief during her first admission. However, it remains unclear why she did not respond or even worsened during her second admission despite receiving the same treatment. One possible explanation is that the initial improvement was primarily due to diazepam’s anxiolytic effects, which may have temporarily reduced anxiety-driven GI symptoms.

On the other hand, patients with Miller Fisher syndrome (MFS), of younger age (<30 years), and having milder disease (e.g., ambulatory) are more likely to experience a recurrence of GBS. Several mechanisms have been proposed to explain this recurrence. One such mechanism involves the FAS gene, which regulates apoptosis and has been linked to persistent immune activation, thereby increasing the risk of recurrence [8].

IVIG remains the first-line treatment for GBS, typically administered at 0.4 g/kg per day for five days [9]. In this case, IVIG led to a rapid resolution of symptoms within 24 hours, reinforcing its efficacy even in atypical GBS presentations. 

## Conclusions

Clinicians should maintain a high index of suspicion for GBS variants in similar cases to avoid diagnostic delays, reduce hospital readmissions, and ensure timely intervention. In such atypical presentations, a trial of IVIG and/or plasmapheresis (to remove harmful antibodies from the blood) should be considered, with close patient monitoring for clinical response. 
